# Duration of lead time in screening for lung cancer

**DOI:** 10.1186/s12890-020-01385-3

**Published:** 2021-01-06

**Authors:** Jochanan Benbassat

**Affiliations:** Department of Medicine (retired), Hadassah Medical Center, PO Box 3894, 91037 Jerusalem, Israel

**Keywords:** Lung cancer, Mass screening, Lead time, Cell doubling time, Chest radiography, Low dose computed radiography

## Abstract

**Background:**

Screening for lung cancer has used chest radiography (CR), low dose computed tomography (LDCT) and sputum cytology (SC). Estimates of the lead time (LT), i.e., the time interval from detection of lung cancer by screening to the development of symptoms, have been derived from longitudinal studies of populations at risk, tumor doubling time (DT), the ratio between its prevalence at the first round of screening and its annual incidence during follow-up, and by probability modeling derived from the results of screening trials.

**Objective:**

To review and update the estimates of LT of lung cancer.

**Methods:**

A non-systematic search of the literature for estimates of LT and screening trials. Search of the reference sections of the retrieved papers for additional relevant studies. Calculation of LTs derived from these studies.

**Results:**

LT since detection by CR was 0.8–1.1 years if derived from longitudinal studies; 0.6–2.1 years if derived from prevalence / incidence ratios; 0.2 years if derived from the average tumor DT; and 0.2–1.0 if derived from probability modeling. LT since detection by LDCT was 1.1–3.5 if derived from prevalence / incidence ratios; 3.9 if derived from DT; and 0.9 if derived from probability modeling. LT since detection of squamous cell cancer by SC in persons with normal CR was 1.3–1.5 if derived from prevalence/incidence ratios; and 2.1 years if derived from the DT of squamous cell cancer.

**Conclusions:**

Most estimates of the LT yield values of 0.2–1.5 years for detection by CR; of 0.9–3.5 years for detection by LDCT; and about 2 years or less for detection of squamous cell cancer by SC in persons with normal CR. The heterogeneity of the screening trials and methods of derivation may account for the variability of LT estimates.

## Background

Screening for lung cancer assumes that the symptomatic illness is preceded by a period of pre-symptomatic disease that is detectable by chest radiography (CR), low dose computed tomography (LDCT) and sputum cytology (SC). The time interval between detection by screening and the onset of clinical manifestations is referred to as lead time (LT). Its duration has been inferred from longitudinal studies of populations at risk, randomized trials of screening for lung cancer, tumor doubling times (DT) and statistical analyzes of the results of screening trials.

Longitudinal studies have either followed prospectively populations at risk by SC [1], or reviewed retrospectively CRs that had been performed before the clinical diagnosis of lung cancer [2]. Estimates based on screening trials have inferred the duration of LT from the ratio between the prevalence of lung cancer at the first (baseline) screening round and the annual incidence of cancer during subsequent follow-up [3]. Inferences from tumor DT assume that a single cell of 10 µm develops into a tumor by a succession of divisions at a constant DT. Therefore, one may derive the duration of LT from the tumor DT and the difference in tumor size at its detection in asymptomatic persons and in symptomatic patients [4]. Statistical analyzes have applied probability estimates on the results of screening trials. The objective of this paper is to search the literature for estimates of the LT of lung cancer and update these estimates by those derived from published screening trials by SC, CR and LDCT.

## Methods

Medline and Old Medline [5] from inception to April 2020 were searched for published estimates of LT of lung cancer, its DT and for reports of screening trials by combining the terms [carcinoma, non-small-cell lung OR carcinoma, small cell OR lung neoplasms] with (a) [mass screening] AND [randomized controlled trials] (117 hits), (b) [sputum] AND [cytology] (215 hits), and (c) [natural history] (132 hits). The reference sections of the retrieved papers were searched for additional relevant studies. Although not a systematic review of additional electronic data stores, this search probably uncovered the main relevant studies, and possible omissions would not have changed the study main conclusions.

## Results

### Longitudinal studies

Prospective serial SC examinations of uranium miners between 1957 and 1970 found sequential abnormalities that consisted of mild atypia followed by moderate atypia, marked atypia and carcinoma in situ [1]. On the average, 2.5 years after detection of carcinoma in situ cells in the sputum, the patients developed invasive carcinoma. 'Invasive carcinoma' was reported to have been diagnosed "cytologically, clinically or at autopsy" [1]; however, the proportion of invasive cancers, which were also radiographically or clinically evident, was not reported.

A 1964 retrospective study of patients with lung cancer, in whom CRs had been made before the definitive diagnosis, revealed an interval between the first CR signs and the first symptoms of 0.8—1 year [2]. Another retrospective study inferred the duration of the LT from the intervals between CR examinations of patients who adhered to the screening protocol (6 months), and the patients who did not (19.5 months). The 13.5 months (= 1.1 years) difference was viewed as an approximation of the interval between CR and clinical detection of lung cancer [6].

### Prevalence/annual incidence ratios

Estimates of LTs derived from prevalence/annual incidence ratios in seven randomized trials of screening by CR ranged between 0.6 and 2.1 years (Table 1). Those derived from controlled randomized trials of screening by LDCT were between 1.1 and 3.5 years (Table 2). Table 3 summarizes LTs derived from randomized trials of screening after selection of cancers detected by SC alone in patients with normal CR. Although comprising only 25% to 40% of all lung cancers, squamous cell lung cancer is practically the only cell type detected by SC in individuals with normal CR. Squamous cell cancer commonly originates centrally and grows as a thin sheet replacing the mucosa, thus escaping radiographic detection while desquamating malignant cells into the sputum [26]. The prevalence / annual incidence ratios of squamous cell lung cancer detected by SC screening only, suggested a LT between cytological and clinical detection of squamous cell lung cancer of 1.2 to 1.8 years.

**Table 1:** Estimated duration of Lead time of lung cancer derived from randomized controlled trials of screening by chest radiography (CR)

Trial and authors	Study population (n)		Prevalence of lung cancers per 1000 at T_0_		Average duration of screening (years)	Average annual incidence of confirmed screen-detected and interval lung cancers per 1000 during screening				Lead time (years)	
	Intervention	Control	Intervention (a)	Control (b)		Intervention, screen-detected (c)	Intervention, interval (d)	Control, screen-detected (e)	Control, interval (f)	a/(c + d)	a/(e + f)
North London: Brett 1968[7]^a^	29,723	25,311	1.0	0.8	3	0.7	0.4	Not applicable	0.8	0.9	1.3
Mayo lung project: Fontana et al. 1984 [8], 1991 [9]^b^	4618	4593	6.8	6.8	6	2.6	1.9	Not applicable	3.2	1.5	2.1
John Hopkins: Frost et al. 1984 [10]; Doria-Rose et al. 2009 [11]^c^	5226	5161	5.4	7.8	7.2	2.6	2.7	3.6	2.9	1.0	0.8
Memorial Sloan Kettering: Melamed et al. 1984 [12]; Doria-Rose et al. 2009 [11]^c^	4968	5072	4.2	4.6	7.2	2.1	2.0	2.4	2.2	1.0	0.9
The Erfurt Trial: Wilde 1989 [13]^d^	41,532	102,348	0.8	0.7	6	0.6	0.8	0.2	0.8	0.6	0.8
Chech Lung Cancer Detection Trial: Kubik et al. 1990 [14], 2000 [15]^e^	3171	3174	3.0	3.0	3	2.9	1.1	Not applicable	2.0	0.8	1.5
PLCO: Oken et al. 2011 [16] ^f^	77,445	77,456	2.3	1.4	3	1.3	0.9	Not applicable	1.7	1.0	1.4

^a^Men, aged 40 years or more. Intervention: Semiannual CR. Control: CR at T0 only

^b^Men, cigarette smokers aged 45 years or more. Intervention: Annual CR and sputum cytology every 4 months. Cancers detected by SC only were excluded from the calculations. Control: Usual care

^c^Men, cigarette smokers aged 45 years or more. Intervention: annual CR and sputum cytology every 4 months. Cancers detected by SC only were excluded from the calculations. Control: Annual CR

^d^Men, aged 40–65. Intervention: Semiannual CR. Control: CR at T0 and at 18 months intervals

^e^Men, heavy cigarettes smokers, aged 40–64 years. Intervention: Semiannual CR and sputum cytology. Control: CR and sputum cytology at T0 only

^f^Entire adult population aged 55–74 years. Intervention: Annual CR. Control: Usual care

**Table 2:** Estimated duration of the lead time of lung cancer derived from randomized controlled trials of screening by Low dose computed tomography (LDCT)

Trial and authors	Study population (n)		Prevalence of lung cancers per 1000 at T_0_		Average duration of screening (years)	Average annual incidence of confirmed screen-detected and interval lung cancers per 1000 during screening				Lead time (years)	
	Interven-tion	Control	Interven-tion (a)	Control (b)		Intervention, screen- detected (c)	Intervention, interval (d)	Control, screen- detected (e)	Control, interval (f)	a/(c + d)	a/(e + f)
Gohagan et al. 2005 [17]^a^	1660	1658	18.1	4.2	1	4.8	1.2	5.4	2.4	3.0	2.3
NLST: Aberle et al. 2013 [18]^b^	26,722	26,732	10.1	5.1	2	7.1	0.7	2.7	2.4	1.3	2.0
DANTE: Infante et al. 2015 [19]^c^	1264	1186	22.9	8.4	4	7.3	7.3	0.4	13.1	1.6	1.7
ITALUNG: Paci et al. 2017 [20]^d^	1613	1593	13.0	1.9	3	4.3	Not given	NA	5.0	3.0	2.6
DLCST: Saghir et al. 2012 [21]^e^	2052	2052	8.3	NA	4	6.3	0.1	NA	2.8	1.3	2.9
LUISI: Becker et al. 2019 [22]^f^	2029	2023	12.8*	2.5*	4	5.4	0.6	NA	3.7*	2.1	3.5
MILD: Sverzellati et al. 2016 [23], Pastorino et al. 2019 [24]^g^	2376	1723	7.2	NA	6.2	3.7	1.8	NA	5.5	1.3	1.3
NELSON: De Koning et al. 2020 [25]^h^	6583	6612	9.0	NA	5.5	4.1	3.9	NA	8.4	1.1	1.1

^a^Individuals aged 55–74 years, current or former smokers. Intervention: Annual LDCT. Control: Annual CR

^b^Adults "at risk". Intervention: Annual LDCT. Control: Annual CR

^c^Male smokers aged 60–74 years. Intervention: Annual LDCT. Control: CR and SC examination at T0 and annual clinical review

^d^Men aged 55–69 years, smokers or former smokers. Intervention: Annual LDCT. Control: Usual care

^e^Heavy smokers / former smokers. Intervention: Annual LDCT. Control: Usual care

^f^Long-term smokers, 50–69 years of age. Intervention: Annual LDCT. Control: Usual care

^g^Smokers current or former, aged 49–75 years. Intervention: Annual or Biannual LDCT. Control: Usual care

^h^Men aged of 50–74 years. Intervention: Biannual LDCT. Control: Usual care

^*^Calculated from data communicated by the authors personally to JB

NA, Not applicable

**Table 3:** Estimated duration of lead time of lung cancer detected by sputum cytology (SC) in patients with normal chest radiography (CR). The prevalenceand incidence of lung cancers detected by SC alone in persons with normal CR were derived from the data reported by the authors

Trial and authors	Study population (n)		Prevalence of squamous cell cancers per 1000 at T_0_		Average duration of screening (years)	Average annual incidence of confirmed squamous cell lung cancers per 1000 during screening				Lead time (years)	
	Intervention	Control	Intervention detected by SC alone (a)	Control detected by SC alone (b)		Intervention, detected by SC alone (c)	Intervention, interval cancers (d)	Control, CR- detected cancers (e)	Control, interval cancers (f)	a/(c + d)	a/(e + f)
Mayo lung project: Fontana et al. 1984 [8], 1991[9]^a^	4618	4593	1.8	6	0.7	0.6*	Not applicable	1.0*	1.3	1.8	
John Hopkins study: Frost et al. 1984 [10]; Doria-Rose et al. 2009 [11]^b^	5226	5161	2.1	Not applicable	7.2	0.9	0.5	0.8	0.9	1.5	1.2
Memorial Sloan Kettering: Melamed et al. 1984 [12]; Doria-Rose et al. 2009 [11]^b^	4968	5072	1.8	Not applicable	7.2	0.8	0.4	0.7	0.8	1.5	1.2

^a^Smokers aged more than 45 years. Intervention: annual CR and sputum cytology every 4 months. Control: Advised to have annual chest radiography and sputum cytology. Intervention and control groups had CR and SC at T0. The prevalence and incidence of lung cancers detected by SC only were derived from published data.

^b^Same as ^A^, except that the controls were offered annual chest radiography.

*Derived from authors' data indicating that 33% of all incidence cancers in the intervention and control group were squamous cell cancers

### Tumor doubling time

The commonly used model of *tumor growth kinetics* assumes an exponential expansion from a single cell at a constant DT that may be estimated by the increase in tumor size on serial CRs [4]. With each doubling of volume, the tumor increases its diameter by approximately 1.26 (the cube root of 2). The average diameter of lung cancer *at clinical diagnosis* is 33 mm (after 35 doublings) [4]; that *at detection by LDCT in asymptomatic persons* is 16 mm (after 32 doublings); and that *at detection by CR* is 30 mm (after 34.5 doublings) [27]. The size *of radiologically occult lung cancer detected by SC,* was estimated by applying the equation of Schwartz [28]:

$${\text{Tumor}}\;{\text{volume}} = \pi /{6}*{\text{ab}}^{{2}}$$

(a = Axial length in mm and b = Approximate average maximal depth in mm).

on the data reported by Woolner et al. [29] in 68 patients with occult lung cancer, and calculated the average volume of their tumors as 135 cmm. For a round tumor of the same volume, this implies a diameter of about 6 mm (after 27.5 doublings). According to this model, one may derive the duration of LT by multiplying the DT of the tumor by the number of doublings needed to reach the average diameter at clinical detection: 7.5 after detection by SC in persons with normal CR; 3 after detection by LDCT; and 0.5 after detection by CR.

The DTs of lung cancer were retrieved from the 2008 review of the literature by Detterbeck and Gibson [27]. They found that clinically detected lung cancers had an average DT of 136 days; lung cancers detected by screening by CR had average DT of 150 days; and lung cancers detected by screening by LDCT had average DT of 480 days. The longer DTs of screen detected cancers is consistent with length bias: screening is more likely to detect selectively slow growing cancers. For squamous cell cancer, the average DT was 104 days for clinical detection; 105 days for detection by screening by CR, and 122 days for detection by screening by LDCT. The respective DTs for adenocarcinoma were 169, 223 and 576 days; and for broncho-alveolar cancer—250, 250 and 764 days.

Therefore, LT of lung cancer would be about 3.9 years after detection by LDCT (three doublings, DT 480 days), 0.2 years after detection by CR (half a doubling, DT 150 days) and 2.1 years after detection by SC in persons with normal CR (7.5 doublings, DT 104 days).

### Probability modeling derived from the results of screening trials

A number of statistical methods have been proposed to derive LT from randomized screening trials for cancer by focusing on screen detected cases and by ignoring interval cases (with LT = 0) [30]. In 2018, Liu et al. [30] used Bayesian posterior samples of key parameters of the NLST-LDCT data to carry out simulations of LT by age and duration of screening intervals. They found that the probability of no-early detection (interval cases) increased with longer screening intervals. Thus, a male heavy smoker had 12% chance of no-early detection with annual screening, and 36% chance with bi-annual screening. The mean LT decreased from 0.9 (standard error—0.7) years with annual screening, to 0.6 (standard error—0.7) years with bi-annual screening.

## Discussion

Derived LTs have fluctuated between 0.2 and 2.1 years since detection by CR 0.9 to 3.9 years since detection by LDCT; and 1.3 and 2.1 years since detection by SC in persons with normal chest x-ray (squamous cell cancer). The modes of LT and its range of probability density curves derived by probability modeling that includes interval cases were 0.24 (0–2.0) years since detection by CR [31], and 0.9 (0–3.0) years since detection by LDCT [30]. Table 4 summarizes the main findings of this survey.

**Table 4:** Estimates of lead time of lung cancer (years) by methods of study

Estimates derived from	From cytological to clinical	From low dose computed tomography to clinical	From radiographic to clinical
Follow up (longitudinal studies)	2.5^a^		0.8–1.1
Prevalence / incidence ratios all histologic types		1.1–3.5	0.6–2.1
Prevalence / incidence ratios squamous cell cancer only	1.3–1.5		1.2–1.8^b^
Doubling time, all histologic types		3.9^c^	0.2^d^
Doubling time, squamous cell lung cancer only	2.1^e^	1.0^f^	0.14^g^
Statistical methods applied on controlled trials (modes and range of probability density curves, and means and standard errors)		Mode: 0.9 (0–3.0) Mean: 0.87 (0.69)	Mode: 0.24 (0–2.0) Mean: 1.0 (1.7)

^a^Time interval between carcinoma in situ and invasive carcinoma

^b^Calculated from the data reported by Doria-Rose et al. 2009 [11] on the prevalence and incidence of squamous cell cancers detected by CR

^c^Assuming a doubling time of 480 days [27]

^d^Assuming a doubling time of 150 days [27]

^e^Assuming a doubling time of 104 days [27]

^f^Assuming a doubling time of 122 days [27]

^g^Assuming a doubling time of 105 days [27]

The main limitations of this study are the methodological differences and possible erroneous assumptions of the approaches to the estimation of LT*.* First, estimates of LT derived from screening trials may have been biased by the heterogeneity in their study populations (see footnotes of Tables 1 and 2). Some trials included men only, while others included men and women; some trials included smokers only, while others included current, former or never smokers. Some trials conducted a single round of follow up examinations, in addition to the baseline round, while other performed several annual examinations. There was also a marked heterogeneity of the histology of the detected lung cancers (data not shown), and histologically different cancers have different LTs.

Second, selection bias may have affected the findings by Saccomanno et al. [1]. As noted by the authors, the restriction of their report to patients who developed invasive carcinoma during the period of observation, may have selected those with a shorter interval between carcinoma in situ to invasive carcinoma. Therefore, the observed 2.5-year interval may have underestimated the time interval between carcinoma in situ and invasive squamous cell cancer.

Third, the estimates of LTs may have been biased by erroneous assumptions when lung cancer becomes radiologically detectable. These assumptions are probably valid for extra-bronchial coin lesions, but not for endobronchial squamous cell cancers that become radiographically evident at a much larger size. Therefore, although LDCT is considered to be the most accurate mass screening modality, it is uncertain whether addition of SC can improve the sensitivity of screening by detecting squamous cell lung cancer before LDCT. It is also questionable whether the equation by Schwartz for extra-bronchial lesions [28] may be applied for occult lung cancer.

A fourth limitation of this study is the uncertainty regarding tumor growth kinetics. On the one hand, a 2018 study that evaluated growth patterns of untreated lung cancer confirmed that the exponential model explains the development of both sub-solid and solid lung cancers [32]. On the other hand, the proliferation curves of almost all animal tumors may be better described by a Gomperzian function [33] that predicts progressively longer DTs as the tumor gets larger, and thereby, a shorter period of preclinical growth than the exponential model, and longer survival after diagnosis.

Both models assume that, if untreated, all cases detected by screening would eventually surface clinically. This assumption is supported by the finding that two thirds of the lung cancer patients detected by SC only [34], and all clinical Stage I lung cancers detected by CR [35] died from lung cancer within 10 years. On the other hand, the estimated average DT of 480 days of cancers detected by screening by LCDT [27] predicts a median survival for lung cancer patients of more than 10 years, rather than the less than a year observed in patents with clinical lung cancer. Therefore, it is possible that LDCT detects mainly slow-growing cancers or tumors, which may not progress to advanced disease [36].

Future research may first, consist of systematic reviews of the literature for screening trials for lung cancer. However, it seems that their heterogeneity with regard to study populations, control populations, and time intervals between screening preclude meta-analysis. Second, SC cytology may be considered again as a screening test, in addition to LDCT. Finally, an effort should be made to resolve the inconsistency between the observed survival of patients with lung cancer detected by LDCT and their calculated LT on the basis of their doubling time.

## Data Availability

Not applicable.

## Abbreviations

CR: Chest radiography
LDCT: Low dose computed tomography
SC: Sputum cytology
LT: Lead time
DT: Tumor doubling time
